# Smith County Medical Society

**Published:** 1902-08

**Authors:** 


### Sabine County Medical Society.

### Smith County Medical Society.

The Smith County Medical Society met in the city of Tyler, Tuesday, July 8th, for which time an interesting program had been arranged.

Dr. J. D. Bryant, in order to attend this meeting, had driven over in a buggy some twelve miles, and brought with him an interesting case for diagnosis. The patient was a boy of eight years, who six months previously had developed a small nodule in the popliteal space, which nodule had gradually increased in size. Of several children all had been healthy. The mother had suffered from sebaccous cysts of the scalp. The tumor in this instance was soft in consistency and gave slight fluctuation. An absolute diagnosis could not be made, but the concensus of opinion was that it was a cystic tumor, either of synovial or sebaceous type, and was located superficial to the deep muscles. There was no bruit, nor pulsation in the tumor.

Dr. T. J. Bell, of Tyler, delegate to the State Medical Association, gave a detailed account of the business transacted by this body, and also gave an outline of the manner advocated for organizing the medical fraternity of Texas. He spoke of the importance of maintaining a strong county medical society.

Dr. J. D. Phillips, of Tyler, read an interesting paper on "Acute Purulent Otitis Media," in which he called attention to the frequent negligence displayed in the management of such cases, both by relatives of the patients and sometimes physicians. In one similar instance the family physician had told the raltives that "the child would outgrow it."

Dr. Albert Woldert, of Tyler, exhibited under the microscope (one-twelfth oil immersion lens) a specimen of zygote of malarial fever, or a malarial parasite of four days' growth within the muscle walls of the middle intestine of a mosquito—Anopheles. This specimen he had succeeded in cultivating while in Philadelphia, in November, 1900, and was the second successful result in America which demonstrated that the malarial parasite, or malarial fever, can be communicated to man by the bite of an infected mosquito—Anopheles. In this case the mosquito had bitten a case of estivo-autumnal fever on Thursday at 8 p. m. and was dissected on the Monday following, with the result that four zygotes were found. He had also succeeded in finding zygotes in a second case of estivo-autumnal fever. The speaker then gave an outline of the work of this kind done by English, German, Italian and American observers, and stated that in investigations to determine the transmission of the disease the "air borne" and "water borne" theories had been ruled out, leaving the mosquito as the avenue through which the disease is communicated to man.

The society then adjourned to meet in Tyler the second Tuesday in August.

THE SECRETARY.

